# Surveillance of Indoor Air Concentration of Volatile Organic Compounds in Luxembourgish Households

**DOI:** 10.3390/ijerph19095467

**Published:** 2022-04-30

**Authors:** Daniel Alvarez-Vaca, Radu Corneliu Duca, Alicia Borras-Santos, Emilie Hardy, Matteo Creta, Carole Eicher, Laurence Wurth, Anne Vergison, An Van Nieuwenhuyse

**Affiliations:** 1 Unit Environmental Hygiene and Human Biological Monitoring, Department of Health Protection, Laboratoire National de Santé (LNS), 1 Rue Louis Rech, L-3555 Dudelange, Luxembourg; daniel.alvarez@lns.etat.lu (D.A.-V.); emilie.hardy@lns.etat.lu (E.H.); matteo.creta@lns.etat.lu (M.C.); 2 Microbiology Department, Laboratoire National de Santé (LNS), 1 Rue Louis Rech, L-3555 Dudelange, Luxembourg; 3 Environment and Health, Department of Public Health and Primary Care, KU Leuven, Kapucijnenvoer 35, 3000 Leuven, Belgium; an.vannieuwenhuyse@lns.etat.lu; 4 Unit Medical Expertise and Data Intelligence, Department of Health Protection, Laboratoire National de Santé (LNS), 1 Rue Louis Rech, L-3555 Dudelange, Luxembourg; alicia.borras@lns.etat.lu; 5 Directorate of Health, Ministry of Health, 13a, rue de Bitbourg, L-1273 Luxembourg, Luxembourg; carole.eicher@ms.etat.lu (C.E.); laurence.wurth@ms.etat.lu (L.W.); anne.vergison@ms.etat.lu (A.V.); 6 Department of Health Protection, Laboratoire National de Santé (LNS), 1 Rue Louis Rech, L-3555 Dudelange, Luxembourg

**Keywords:** indoor pollution, indoor air quality, volatile organic compounds, risk assessment

## Abstract

Exposure to air pollution is a well-known health risk. For instance, volatile and very volatile organic compounds (VOCs and VVOCs) are known to cause respiratory, haematologic or immune diseases, and even cancer. Based on the Luxembourgish indoor pollution surveillance program, we performed an exploratory analysis for the period 2014–2019, in order (1) to evaluate the prevalence of VOCs and VVOCs in households, and (2) to estimate the risks of lifelong exposure to selected VOCs on the health of the adult population. The database included 715 indoor air samples from 159 different households. Observed VOC and VVOC levels were similar to those in neighbouring countries. Our health impact assessment identified some health risks associated with the observed concentrations in Luxembourg. Furthermore, this study shows the major public health importance of having a national indoor pollution surveillance system in place. Highlights: (1) This study provides an overview of the domestic indoor pollution in Luxembourg. (2) (V)VOCs levels in Luxembourg were similar to those in neighbouring countries. (3) The results clearly show the importance of having a surveillance system in place.

## 1. Introduction

Exposure to air pollution is a well-known health risk. Depending on the compounds present, their concentration and their toxicity, different symptoms may arise after acute exposure, either topical (e.g., irritation of skin, eyes, nose or throat) or systemic (e.g., headache, dizziness, asthenia or nausea) [1]. Moreover, chronic exposure to air pollution is a well-known risk factor for developing chronic diseases; certain volatile and very volatile organic compounds, (V)VOCs, are known to cause respiratory, haematological or immune diseases, as well as cancer [2,3,4,5]. The World Health Organisation (WHO) estimates that air pollution is the main contributor to environmental health risks, causing an annual estimation of 6 million premature deaths globally [6] and, specifically in the WHO European region (WHO-Eu), between 47 and 106 deaths per 100,000 inhabitants (high and low–middle income, respectively) [7]. Previous studies in Europe showed that the population spends around 90% of its time indoors, and more than 70% of it at home [8,9,10]. Therefore, the study of household air pollution is of great interest in public health. According to a regional study by the WHO-Eu, household air pollution is estimated to cause between 3 and 37 deaths per 100,000 (high and low–middle income, respectively; between 17,700 and 99,500 total annual deaths) [7].

Air pollution mainly includes particulate matter, inorganic gases and (V)VOCs, which are the focus of this study. (V)VOCs can be released into indoor air from furniture, carpets, housing products, dry-cleaning products, paints, organic-fuelled heating and cooking systems [11,12]. Environmental tobacco smoke and garages attached to households may also be a source of indoor air pollution [13]. Additionally, (V)VOCs can enter indoor environments from outdoor anthropological sources such as traffic, industry, agricultural activities and waste treatment plants, as well as natural sources such as emissions from plants [12].

In recent years, household air pollution has been widely studied, analysing the concentration of different air pollutants in Europe [9,14] and assessing their health risks [14,15,16]. However, few data are available about indoor pollution in the Grand Duchy of Luxembourg. To the best of our knowledge, only one article has been recently published studying pesticides in one household located in Luxembourg city [17].

Lastly, within the Grand Duchy of Luxembourg, population and economical activities are distributed unevenly. The industrial sector (mainly mining and steel industry) dominates the south-west, where a higher population density can be found, whilst the remaining peripheral area focuses on agricultural activities and is less densely populated [18,19]. The canton of Luxembourg is the most densely populated, and it is the traffic nerve centre of the country [20]. All these factors may entail a differential distribution of air pollution sources, which needs to be analysed.

In this context, we performed an exploratory analysis for the period 2014–2019 in order to evaluate the prevalence of the selected (V)VOCs in households, and to estimate the risks of lifelong exposure to selected (V)VOCs on the health of the adult population.

## 2. Materials and Methods

### 2.1. Sample Collection

A total of 715 indoor air samples (370 general VOCs and 345 VVOCs) were collected from 159 different households in the Grand Duchy of Luxembourg from 2014 to 2019. They were collected as part of the national indoor pollution surveillance programme by the Unit Environmental Hygiene and Human Biological Monitoring, Laboratoire National de Santé (Dudelange, Luxembourg). This programme foresees that physicians can request an investigation of the patient’s residence, in the case of health complaints for which they suspect a potential cause in the indoor environment. After keeping windows closed for 24 h and a normal use of the premises, air sampling pumps Gilair Plus (Gilian, St. Petersburg, FL, USA) were placed in the most frequented rooms (i.e., sleeping room, living room, kitchen, office, etc.), at a central position and a medium height. For VVOCs, a cartridge impregnated with dinitrophenylhydrazine (DNPH) was used, Supelco LpDNPH S10 (Supelco Inc, Bellefonte, PA, USA), and an air volume of 20 L was sampled. For VOCs, Tenax tubes were used and an air volume of 2 L was sampled. Sampling of VVOCs and VOCs was carried out following the ISO 16000-3:2011 and ISO 16000-6:2011 recommendations, respectively.

### 2.2. Chemical Analyses and Reagents

VOC levels were analysed by thermal desorption (TD100 Markes) coupled to a gas chromatograph (GC 6890N, Agilent Technologies: Diegem, Belgium)–mass spectrometer (MS 5973i, Agilent Technologies: Diegem, Belgium). VVOCs were analysed after elution with acetonitrile on a high-performance liquid chromatography system (HPLC 1260, Agilent Technologies: Diegem, Belgium) with a diode-array detector (DAD VL+ Agilent). All the samples were analysed in the same laboratory for the entire period of the survey and quality controls were systematically performed following the ISO 16000-3:2011 and ISO 16000-6:2011 recommendations. GLIMS software (CliniSys-MIPS, Ghent, Belgium) was used as a laboratory information system.

### 2.3. Statistical Analysis

The normal distribution of compound concentrations was studied first, both graphically (by boxplots) and statistically (by Shapiro–Wilk test). Due to high right-skewness, the Shapiro–Wilk test was used on log-transformed concentrations, but the normal hypothesis was rejected. Therefore, the results from chemical analyses were summarised using the geometrical mean (GM) and geometric standard deviation. Complementary quantile distribution measures were also calculated. All compounds were classified according to their compliance with guidance values provided by the German Association of Environmental Institutes (AGOEF) and the German Federal Environmental Agency (Umweltbundesamt) [21]. Compounds showing levels above the guidance values at the 75th percentile were selected, and additional compounds of environmental and health relevance were also included. Co-exposure to selected compounds was also assessed, using Spearman’s correlation.

Spatial analysis was based on three environmental regions: urban background influenced by traffic (UBT), urban background influenced by industries (UBI) and rural background (RB), corresponding to Luxembourg canton, Esch-sur-Alzette canton and the remaining cantons, respectively. Differences in concentrations among regions were studied using the non-parametrical Kruskal–Wallis test, due to a lack of normality. Compounds showing statistically significant *p*-values (*p* ≤ 0.05) were subjected to a post hoc pairwise analysis via Mann–Whitney U test, and *p*-values were corrected using Holms method.

Temporal analysis was performed using linear regression on concentrations and year of sampling.

All statistical analyses were performed using R v4.0.3 (R Foundation for Statistical Computing: Vienna, Austria) and RStudio v1.4.1103 (RStudio, PBC: Boston, MA, USA).

### 2.4. Risk Assessment

Finally, a risk assessment on carcinogenic effects was performed for lifelong exposure to the selected pollutants. The methodology proposed by the United States Environmental Protection Agency (US EPA) was observed [22], using the following equations:

(1) $$LCR_{i}=EC_{i}\times IUR_{i}$$

(2) $$LCR=\overset{}{\underset{}{\sum }}LCR_{i}$$

where *LCR_i_* is the estimated individual risk for lifelong (70 years) exposure to pollutant *i* (unitless), *EC_i_* the estimated exposure to pollutant *i* (μg m^−3^), and *IUR_i_* the inhalation unit risk for pollutant *i* (μg^−1^ m^3^). *IUR* is defined by the US EPA as the “upper-bound excess lifetime cancer risk estimated to result from continuous exposure to an agent via inhalation per μg/m^3^ over a lifetime” [22] (12-3). *IUR* values published by the California Office of Environmental Health Hazard Assessment (OEHHA) were used [3]. Cumulative risk from all carcinogenic pollutants was also calculated (Equation (2)).

Additionally, non-carcinogenic risk was also estimated calculating the hazard quotient (HQ) for each compound and target-organ-specific hazard index (TOSHI), as proposed by the US EPA [22]:

(3) $$HQ_{i}=\frac{EC_{i}}{RfC_{i}}$$

(4) $$TOSHI_{j}=\overset{}{\underset{}{\sum }}HQ_{j}$$

where *HQ_i_* is the estimated non-carcinogenic risk for pollutant *i* (unitless) and *RfC_i_* the reference concentration for pollutant *i* (μg m^−3^). The US EPA defines the *RfC* as an “estimate of continuous inhalation exposure that is likely to be without an appreciable risk of deleterious effects during a lifetime” [22] (12-3). *RfC* values were also obtained from the OEHHA [23]. Cumulative risk for all pollutants affecting the same target organ was calculated (Equation (4)).

## 3. Results

### 3.1. Summary of Exposure Measurements

An overview of the concentrations found by compound is shown in Table 1. A stratified selection was carried out according to guidance values, including those compounds exceeding their guidance values in 25% of samples or higher: methylisothiazolinone (MIT) (at 50th percentile), and benzene, formaldehyde, limonene and β-pinene (at 75th percentile). MIT only started to be analysed in 2016, and therefore fewer samples of it could be studied. Additionally, another 11 known hazardous pollutants were included for further analysis. The concentrations for all 51 compounds analysed can be found in the Supplementary Material (Table S1).

Co-exposure among selected compounds was assessed by Spearman’s correlation. A strong positive association was observed among the three xylenes (r_s_ > 0.90, *p* < 0.001) and between them and ethyl-benzene (r_s_ > 0.89, *p* > 0.1). Otherwise, a low-to-moderate positive correlation was estimated generally, with the least correlated compounds being ethoxyethoxyethanol and trichloroethylene (mean r_s_ = 0.01 for both).

### 3.2. Spatial Analysis

The Kruskal–Wallis test for comparing concentrations among environmental regions only showed statistically significant differences (*p* ≤0.05) for aromatic hydrocarbons (see Table 2). Within this group, the rural region generally presented the highest mean values, as seen for xylenes, styrene and propyl-benzene, whereas the urban region influenced by traffic generally presented the lowest mean values, with the strongest significant differences for propyl-benzene and naphthalene (*p* <0.001). The urban region influenced by industry presented a significantly higher level for benzene (*p* = 0.014).

Regarding other chemical categories, butoxypropanol presented higher values in the industrial region and lower values in the traffic-related region (*p* = 0.054). No significant difference was found for other compounds.

### 3.3. Temporal Analysis

In order to assess temporal variation, a linear regression model was estimated for year of sampling (as the independent variable) and compound concentration (as the dependent variable). Log-transformed concentration was used due to right skewness. For compounds with a significant slope estimation (b estimate), relative variation was also calculated according to the geometric mean (GM) and guidance values, as shown in Table 3.

No compound showed a significant increase, according to the model fit. On the contrary, most of the selected compounds showed a decreasing trend. The strongest relative variations were found for perchloroethylene (−0.89 μg/m^3^/year, −88.5% of its guidance value) and methylisothiazolinone (−0.62 μg/m^3^/year, −61.9% of its guidance value). The remaining compounds with significant variation showed decreases that accounted for less than 10% of their guidance values.

### 3.4. Risk Assessment

The estimated risk derived from lifelong exposure to hazardous air pollutants is shown in Table 4.

Regarding cancer risk, naphthalene has the highest estimated inhalation unit risk (*IUR*), followed by benzene and 1,4-dichorobenzene. Accordingly, naphthalene presented the highest estimated risk (148 expected cancer cases per 1 million people exposed to the GM concentration), followed by benzene (79 expected cancer cases), whilst the estimated risk for 1,4-dichlorobenzene is much lower (three expected cancer cases) due to its low measured concentration. On the other hand, formaldehyde has a lower *IUR* than the former, but an estimated risk similar to benzene (71 expected cancer cases), as exposure to this compound is generally higher. Globally, cancer risk after lifelong exposure to all compounds studied is estimated in 335-1162 expected cancer cases per 1 million people exposed to GM or the 90th percentile concentration, respectively.

Regarding non-cancer effects, naphthalene and formaldehyde present the highest risks (the lowest reference concentration), but only the former is estimated to be a risk at the concentrations measured (hazard quotient of 1.3 at GM concentration and 6.2 at 90th percentile), the respiratory tract being its main target organ. When assessing the risk for target organs after exposure to all related compounds, only the respiratory tract is estimated to be at risk (hazard index of 1.4 at GM concentration and 6.4 at 90th percentile), majorly by exposure to formaldehyde. All remaining compounds assessed were estimated to be of very low risk (0 to 0.2 hazard quotient) for non-cancer chronic health effects.

## 4. Discussion

This study provides an overview of the current situation with regard to domestic indoor pollution in the Grand Duchy of Luxembourg. Measurements gathered over 5 years were included, allowing us to make more solid estimations and to reduce potential biases from short-term variations. Moreover, the inclusion of measurements from different environmental regions enabled a further characterisation of exposure from different backgrounds. The sample selection, being based on medical prescription, allowed a more conservative approach, studying the population that is expected to be at higher risk. However, this might lead to the overestimation of pollutant concentrations, and this must be kept in mind when interpreting the results.

High levels were detected for several compounds, especially MIT, but also benzene, formaldehyde, limonene and β-pinene. To the best of our knowledge, there are no studies assessing MIT levels in household indoor air.

Regarding the remaining compounds of study, their concentrations are mostly comparable to those found in other indoor air studies in European cities [9,14,16]. Measurements for benzene are similar to those from neighbouring countries (GM 1.5–3.2 μg/m^3^), as well as those for napthalene (GM 0.8 μg/m^3^), styrene (GM 1.4–3.9 μg/m^3^) and xylenes (GM 0.8–9.8 μg/m^3^) [14,16]. Measurements for formaldehyde remain lower than measurements made in France (GM 20.0–33.5 μg/m^3^), Germany (GM 36.0 μg/m^3^) and Spain (GM 54.6 μg/m^3^) [14,16]. Limonene shows high variability between European cities, with Luxembourg’s mean levels being similar to Belgium’s (GM 10.6 μg/m^3^), but higher than the Netherlands’ (GM 4.1–6.4 μg/m^3^) and lower than Germany’s (GM 2.4–32.9 μg/m^3^) [14]. Finally, the measurements included in this study were below the guidance values of the WHO-Eu [5,10].

With respect to the geographical distribution of pollutants in the Grand Duchy of Luxembourg, in this study, households from urban settings influenced by traffic were found to have the lowest mean levels of aromatic hydrocarbons, whereas households from rural areas generally showed the highest values, followed by those from industrial areas. Cirillo et al. [24] described a similar differential distribution for polycyclic aromatic hydrocarbons in Italy, with higher levels in rural areas compared with urban settings, and discussed its possible link to heating by fireplaces. Similarly, the usage of fuel-oil-dependent heating systems could be associated with this pollution pattern in the Grand Duchy of Luxembourg. These systems are broadly spread in rural regions of the country (about 70% of households), whereas urban areas rely mainly on natural gas (only 17% of urban households use fuel oil) [25].

Regarding the temporal analysis performed in this study, a global decreasing trend was found. MIT, which was the compound that most exceeded the guidance values, was also the one to show the greatest reduction during the period of study, along with perchloroethylene (according to logged slope and relative variation, respectively). A generalised improvement in air quality over the last few decades was also described by the European Environment Agency (EEA) [26], who associates this decrease in air pollutants with specific legislation. Particularly in indoor settings, the EEA observed great reductions following the approval of smoking restrictions [11].

Finally, risk assessment was also an important part of this study. The highest cancer risk was identified for exposure to naphthalene, benzene and formaldehyde, whereas non-cancer risk was only found for formaldehyde exposure, especially for the nervous system. Previous studies have also estimated the risk from exposure to indoor air pollution, but comparison among them is not straightforward due to the usage of older *IUR* and reference concentration values, different selections of pollutants and methodology variations. Payne-Sturges et al. [15] used lower *IUR* values for benzene and ethylbenzene (respectively, 7.8 × 10^−6^ and 5.0 × 10^−7^), estimating lower cancer risks for concentrations that were similar to or higher than ours (respectively, GM 3.7 and 3.2 μg/m^3^). Huang et al. [27] and Dai et al. [28] adapted the risk estimation to the time spent indoors by the Chinese population, therefore obtaining lower risks than the standard US EPA methodology [22]. However, their estimations for the time spent indoors are similar to those made by previous studies in Europe (90%) [8,9,10]. Estimates of cumulative cancer risk directly depend on the number and selection of pollutants included, so a comparison between studies analysing a different combination of pollutants is not possible. Nevertheless, as risk estimation is based on levels of exposure, and the concentrations found in our study are similar to those in previous European studies referenced above, it can be inferred that a similar risk is to be expected. According to Hänninen et al. [29], Luxembourg has a similar burden of disease induced by VOC exposure than the Netherlands or Sweden, and lower than Belgium, Germany and France.

## 5. Conclusions

Our health impact assessment has identified some health risks associated with the observed concentrations. As observed (V)VOC levels in Luxembourg were similar to those in neighbouring countries, the identified health risk will also be comparable. The results clearly show the importance of having such a surveillance system in place to (i) support the prescribing physicians in patient management and care, (ii) define public health priorities for Luxembourg in the domain of indoor pollution and better targeted prevention, and to (iii) identify niches for focused science-policy research.

## Figures and Tables

**Table 1:** Overview of a selection of hazardous air pollutants, by quantile of compliance with guidance values.

Category	Compound (μg/m^3^)	n	GM	GSD	Min	p10	p25	p50	p75	p90	Max	Guidance Values *	IARC
<p50													
Other VOCs	methylisothiazolinone	232	1.2	2.2	0.5	0.5	0.5	1.0	2.0	4.0	9.5	<1.0	–
<p75													
Acyclic aliphatic aldehydes	formaldehyde	345	11.9	6.1	0.5	0.5	8.6	24.9	40.1	55.5	368.8	30.0	1
Aromatic hydrocarbons	benzene	369	2.7	2.2	0.2	1.0	1.5	2.5	4.0	8.0	37.0	3.0	1
Terpenes	limonene	370	10.3	4.3	0.2	1.0	4.5	10.0	26.2	81.7	605.0	23.0	3
Terpenes	pinene, β-	370	3.8	5.0	0.1	1.0	1.5	4.0	10.0	29.4	626.0	8.7	–
<p90													
Acyclic aliphatic aldehydes	acetaldehyde	338	9.9	6.3	0.5	0.5	4.7	17.7	32.1	57.1	342.8	54.0	2B
Aliphatic hydrocarbons	hexane, n-	370	2.5	3.6	0.2	0.3	1.0	2.5	5.0	15.9	941.5	8.0	–
Aromatic hydrocarbons	ethyl-benzene	370	2.1	3.2	0.2	1.0	1.0	1.5	3.0	14.1	100.5	10.0	2B
Aromatic hydrocarbons	naphthalene	370	0.6	3.1	0.1	0.1	0.1	1.0	1.0	1.5	18.0	1.2	2B
Aromatic hydrocarbons	xylene, m-	370	3.8	3.9	0.2	1.0	1.5	2.5	7.0	33.8	269.0	29.0	3
Aromatic hydrocarbons	xylene, o-	370	2.2	3.6	0.2	1.0	1.0	1.5	3.5	16.7	154.3	9.0	3
Halocarbons	dichlorobenzene, 1,4-	370	0.3	1.9	0.2	0.2	0.2	0.2	0.2	1.0	30.0	<1.0	2B
Halocarbons	perchlorethylene	370	0.2	3.9	0.1	0.1	0.1	0.1	0.5	1.0	464.5	<1.0	2A
>p90													
Aromatic hydrocarbons	styrene	370	1.7	2.6	0.2	1.0	1.0	1.0	2.5	5.5	3872.0	12.0	2A
Aromatic hydrocarbons	xylene, p-	370	1.8	3.3	0.2	0.5	1.0	1.0	3.0	11.5	119.0	29.0	3
Halocarbons	trichloroethylene	370	0.3	1.5	0.2	0.2	0.2	0.2	0.2	0.2	5.0	<1.0	1

Abbreviations: VOCs: volatile organic compounds; n: number of samples; GM: geometric mean; GSD: geometric standard deviation; p10–p90: percentiles; IARC: classification by the International Agency for Research on Cancer. * Guidance values according to the German Association of Environmental Institutes (AGÖF).

**Table 2:** Concentration of selected hazardous air pollutants, by region.

Compound (μg/m^3^)	RB		UBI		UBT		*p* Value	Pairwise		
	N	GM (GSD)	N	GM (GSD)	N	GM (GSD)		RB-UBI	RB-UBT	UBI-UBT
Aromatic hydrocarbons										
benzene	206	2.76 (2.31)	71	3.12 (2.04)	92	2.34 (1.93)	0.014	0.084	0.238	0.005
ethyl-benzene	207	2.39 (3.46)	71	1.95 (3.02)	92	1.55 (2.62)	0.086	–	–	–
propyl-benzene, i-	207	0.40 (3.08)	71	0.38 (3.03)	92	0.23 (2.32)	<0.001	0.829	<0.001	0.002
naphthalene	207	0.62 (3.00)	71	0.72 (3.38)	92	0.39 (2.97)	<0.001	0.293	0.001	0.001
styrene	207	1.89 (2.95)	71	1.41 (2.27)	92	1.45 (2.09)	0.023	0.133	0.040	0.722
xylene, m-	207	4.48 (4.34)	71	3.47 (3.50)	92	2.73 (3.14)	0.073	–	–	–
xylene, o-	207	2.60 (3.86)	71	1.87 (3.57)	92	1.72 (3.02)	0.048	0.185	0.084	0.837
xylene, p-	207	2.08 (3.49)	71	1.63 (3.43)	92	1.29 (2.75)	0.014	0.431	0.011	0.431
Aliphatic hydrocarbons										
hexane, n-	207	2.68 (3.58)	71	2.27 (3.91)	92	2.31 (3.35)	0.356	–	–	–
Terpenes										
limonene	207	10.64 (4.41)	71	10.82 (4.97)	92	9.35 (3.74)	0.608	–	–	–
pinene, β-	207	4.00 (4.34)	71	4.32 (5.69)	92	3.13 (5.92)	0.198	–	–	–
Esters of alcohols										
butoxypropanol	207	2.11 (4.73)	71	2.84 (5.11)	92	1.57 (3.95)	0.054	–	–	–
ethoxyethoxyethanol	207	1.54 (3.57)	71	1.22 (4.63)	92	1.90 (2.93)	0.171	–	–	–
Halocarbons										
dichlorobenzene, 1,4-	207	0.31 (1.83)	71	0.34 (1.90)	92	0.30 (1.87)	0.133	–	–	–
perchlorethylene	207	0.20 (3.99)	71	0.21 (4.28)	92	0.22 (3.48)	0.505	–	–	–
trichloroethylene	207	0.27 (1.44)	71	0.26 (1.26)	92	0.28 (1.62)	0.769	–	–	–
Other VOCs										
methylisothiazolinone	121	1.25 (2.33)	61	1.00 (2.18)	50	1.13 (2.09)	0.215	–	–	–
Acyclic aliphatic aldehydes										
formaldehyde	185	13.50 (5.80)	67	11.41 (6.47)	93	9.56 (6.40)	0.193	–	–	–
acetaldehyde	182	11.63 (6.33)	66	7.71 (5.75)	90	8.56 (6.65)	0.105	–	–	–
Other aldehydes										
tolualdehyde	151	0.51 (1.34)	55	0.50 (1.00)	76	0.53 (1.53)	0.671	–	–	–

Abbreviations: N: number of samples; GM: geometric mean; GSD: geometric standard deviation; VOCs: volatile organic compounds. Regions: rural background (RB), urban background influenced by industry (UBI), and urban background influenced by traffic (UBT).

**Table 3:** Temporal analysis of concentrations for selected hazardous air pollutants.

Compound (µg/m^3^)	Log-Linear Regression Estimates			Relative Variation			
	b	Unlogged b	*p*-Value	GM	GM %	GV	GV %
Aromatic hydrocarbons							
benzene	0.01	1.01	0.555	–	–	–	–
ethyl-benzene	−0.15	−0.86	<0.001	2.1	−41.9%	10.0	−8.6%
naphthalene	−0.04	−0.96	0.293	–	–	–	–
styrene	−0.15	−0.86	<0.001	1.7	−51.1%	12.0	−7.1%
xylene, m-	−0.20	−0.81	<0.001	3.8	−21.6%	29.0	−2.8%
xylene, o-	−0.16	−0.85	<0.001	2.2	−38.5%	9.0	−9.4%
xylene, p-	−0.14	−0.87	<0.001	1.8	−49.1%	29.0	−3.0%
Aliphatic hydrocarbons							
hexane, n-	−0.25	−0.78	<0.001	2.5	−31.1%	8.0	−9.7%
Terpenes							
limonene	0.07	1.07	0.129	–	–	–	–
pinene, β-	0.04	1.04	0.447	–	–	–	–
Halocarbons							
dichlorobenzene, 1,4-	−0.01	−0.99	0.670	–	–	–	–
perchlorethylene	−0.12	−0.89	0.003	0.2	−425.3%	<1.0	−88.5%
trichloroethylene	0.01	1.01	0.206	–	–	–	–
Other VOCs							
methylisothiazolinone	−0.48	−0.62	<0.001	1.2	−53.7%	<1.0	−61.9%
Acyclic aliphatic aldehydes							
formaldehyde	−0.28	−0.75	<0.001	11.9	−6.3%	30.0	−2.5%
acetaldehyde	−0.36	−0.70	<0.001	9.9	−7.1%	54.0	−1.3%

b: log-linear regression estimate; GM: geometric mean; GV: guidance value; VOCs: volatile organic compounds.

**Table 4:** Risk estimation for a selection of household air pollutants.

	Concentration (µg/m^3^)		Cancer Risk			Non-Cancer Risk			
Compound			*IUR* (m^3^/µg)	Excess Cases *		*RfC* (µg/m^3^)	Hazard Quotient		Target Organs and Processes
	GM	90th		GM	90th		GM	90th	
Aromatic hydrocarbons									
benzene	2.7	8	2.9 × 10^−5^	79	232	60	0.0	0.1	DEV, HAE, NER
ethyl-benzene	2.1	14.1	2.5 × 10^−6^	5	35	–	–	–	–
naphthalene	0.6	1.5	2.6 × 10^−4^	148	390	9	0.1	0.2	RES
styrene	1.7	5.5	–	–	–	900	0.0	0.0	NER
xylenes	7.7	62	–	–	–	700	0.0	0.1	NER, OPH, RES
Aliphatic hydrocarbons									
hexane, n-	2.5	15.9	–	–	–	7000	0.0	0.0	NER
Halocarbons									
dichlorobenzene, 1,4-	0.3	1	1.1 × 10^−5^	3	11	–	–	–	–
perchlorethylene	0.2	1	6.1 × 10^−6^	1	6	–	–	–	–
trichloroethylene	0.3	0.2	2.0 × 10^−6^	1	0	600	0.0	0.0	NER, OPH
Acyclic aliphatic aldehydes									
formaldehyde	11.9	55.5	6.0 × 10^−6^	71	333	9	1.3	6.2	NER
acetaldehyde	9.9	57.1	2.7 × 10^−6^	27	154	–	–	–	–

GM: geometric mean; 90th: 90th percentile; *IUR*: inhalation unit risk; *RfC*: reference concentration. Target organs and processes: DEV—developmental effects, HAE—haematological system, NER—nervous system, OPH—ophthalmological effects, RES—respiratory tract. “–”: unknown value. * Excess cases per 1 million people exposed.

## Data Availability

The aggregated data presented in this study are available in the Supplementary Material.

## Supplementary Materials

The following supporting information can be downloaded at: https://www.mdpi.com/article/10.3390/ijerph19095467/s1, Table S1: Overview of studied VOCs and VVOCs (µg/m^3^), by quantile of compliance with guideline values.
