# Supplementary material for: Surveillance of Indoor Air Concentration of Volatile Organic Compounds in Luxembourgish Households
Source: Int J Environ Res Public Health. 2022 Apr 30;19(9):5467. doi: 10.3390/ijerph19095467 (PMC9105303; doi:10.3390/ijerph19095467)
Supplement: Supplementary file 1 [file ijerph-19-05467-s001.zip › ijerph-1687478-supplementary.pdf]

Supplementary Material

Table S1. Overview of studied VOCs and VVOCs (μg/m³), by quantile of compliance with guideline values.

Category	Compound (μg/m³)	n	GM	GSD	min	p10	p25	p50	p75	p90	Max	Ref. Value
50th percentile												
Other VOCs	methylisothiazolinone	232.0	1.2	2.2	0.5	0.5	0.5	1.0	2.0	4.0	9.5	<1.00
75th percentile												
Acyclic aliphatic aldehydes	formaldehyde	345.0	11.9	6.1	0.5	0.5	8.6	24.9	40.1	55.5	368.8	30.0
Aromatic hydrocarbons	benzene	369.0	2.7	2.2	0.2	1.0	1.5	2.5	4.0	8.0	37.0	3.0
Terpenes	limonene	370.0	10.3	4.3	0.2	1.0	4.5	10.0	26.2	81.7	605.0	23.0
	pinene, β-	370.0	3.8	5.0	0.1	1.0	1.5	4.0	10.0	29.4	626.0	8.7
90th percentile												
Acyclic aliphatic aldehydes	acetaldehyde	338.0	9.9	6.3	0.5	0.5	4.7	17.7	32.1	57.1	342.8	54.0
	nonanal	321.0	2.8	5.3	0.5	0.5	0.5	7.2	13.4	19.0	32.6	19.0
Aliphatic hydrocarbons	hexane, n-	370.0	2.5	3.6	0.2	0.3	1.0	2.5	5.0	15.9	941.5	8.0
	heptane, n-	370.0	2.7	2.8	0.2	1.0	1.0	2.0	5.0	9.5	130.5	9.0
	octane, n-	370.0	2.2	3.0	0.2	1.0	1.0	1.6	4.1	10.0	102.0	5.0
	nonane, n-	370.0	2.2	3.7	0.2	0.5	1.0	1.5	4.5	16.3	251.5	5.0
	decane, n-	370.0	2.1	4.1	0.2	0.2	1.0	1.5	5.5	15.5	334.5	11.0
	undecane, n-	370.0	2.0	4.5	0.1	0.1	1.0	1.6	5.5	14.0	180.0	14.0
Aromatic hydrocarbons	ethyl-benzene	370.0	2.1	3.2	0.2	1.0	1.0	1.5	3.0	14.1	100.5	10.0
	propyl-benzene, n	370.0	0.8	4.1	0.1	0.1	0.5	1.0	1.5	6.0	42.0	2.1
	naphthalene	370.0	0.6	3.1	0.1	0.1	0.1	1.0	1.0	1.5	18.0	1.2
	toluene	370.0	8.7	3.7	0.2	2.0	3.0	7.0	19.6	67.5	340.5	30.0
	trimethylbenzene, 1,2,3-	370.0	0.9	4.0	0.1	0.1	0.5	1.0	1.5	5.0	38.0	2.6
	trimethylbenzene, 1,2,4-	370.0	2.9	3.7	0.2	1.0	1.0	2.0	5.5	22.4	182.6	11.0
	trimethylbenzene, 1,3,5-	370.0	0.8	4.2	0.1	0.1	0.5	1.0	1.5	6.0	53.3	3.0
	xylene, m-	370.0	3.8	3.9	0.2	1.0	1.5	2.5	7.0	33.8	269.0	29.0
	xylene, o-	370.0	2.2	3.6	0.2	1.0	1.0	1.5	3.5	16.7	154.3	9.0
Esters of alcohols	phenoxyethanol	232.0	2.0	2.9	0.5	0.5	0.5	2.0	4.9	8.0	19.5	5.0
Halocarbons	dichlorobenzene, 1,4-	370.0	0.3	1.9	0.2	0.2	0.2	0.2	0.2	1.0	30.0	<1.00
	perchlorethylene	370.0	0.2	3.9	0.1	0.1	0.1	0.1	0.5	1.0	464.5	<1.00
Maximum value												
Acyclic aliphatic aldehydes	propanal	284.0	0.7	2.7	0.5	0.5	0.5	0.5	0.5	0.5	241.7	14.0
	butanal	282.0	0.6	2.5	0.5	0.5	0.5	0.5	0.5	0.5	138.8	10.0
	pentanal	285.0	0.8	3.5	0.5	0.5	0.5	0.5	0.5	9.9	353.1	20.0
	hexanal	309.0	3.2	7.0	0.5	0.5	0.5	4.6	16.7	33.3	806.3	55.0
	heptanal	282.0	0.6	1.8	0.5	0.5	0.5	0.5	0.5	0.5	41.1	6.7
	octanal	282.0	0.6	1.8	0.5	0.5	0.5	0.5	0.5	0.5	28.1	8.0
	decanal	282.0	0.6	2.0	0.5	0.5	0.5	0.5	0.5	0.5	18.5	7.0
Aliphatic hydrocarbons	dodecane, n-	370.0	1.5	3.5	0.1	0.1	1.0	1.5	3.5	7.0	64.0	9.0
	tridecane, n-	370.0	1.1	2.6	0.2	0.2	1.0	1.0	1.5	3.5	103.0	5.0

	tetradecane, n-	370.0	1.4	2.7	0.2	0.2	1.0	1.5	2.5	3.5	26.5	4.0
	styrene	370.0	1.7	2.6	0.2	1.0	1.0	1.0	2.5	5.5	3872.0	12.0
	xylene, p-	370.0	1.8	3.3	0.2	0.5	1.0	1.0	3.0	11.5	119.0	29.0
Esters of alcohols	butylacetate	232.0	4.3	3.8	0.5	0.5	1.5	4.8	11.5	23.6	327.8	27.0
	butoxyethanol	370.0	1.9	5.7	0.1	0.1	1.0	2.0	4.5	11.0	5916.5	13.0
	butoxyethoxyethanol	370.0	1.5	3.1	0.2	0.2	1.0	1.5	3.0	6.5	25.5	8.0
Halocarbons	trichloroethylene	370.0	0.3	1.5	0.2	0.2	0.2	0.2	0.2	0.2	5.0	<1.00
Ketones	butanone	184.0	0.7	3.2	0.5	0.5	0.5	0.5	0.5	8.8	58.6	33.0
Other aldehydes	crotonaldehyde	179.0	0.5	1.4	0.5	0.5	0.5	0.5	0.5	0.5	34.4	<2.00
	methacrolein	179.0	0.5	1.3	0.5	0.5	0.5	0.5	0.5	0.5	13.6	<3.00
	benzaldehyde	282.0	0.6	2.0	0.5	0.5	0.5	0.5	0.5	0.5	32.1	15.0
Terpenes	carene, 3-	370.0	1.7	4.9	0.1	0.1	1.0	1.5	4.5	15.5	211.5	26.0
	pinene, α-	370.0	4.9	4.3	0.1	1.0	2.0	4.5	12.0	34.8	396.0	68.0
No guideline value												
Aromatic hydrocarbons	propyl-benzene, i-	370.0	0.3	3.0	0.1	0.1	0.1	0.1	1.0	1.5	10.9	–
Esters of alcohols	butoxypropanol	370.0	2.1	4.6	0.2	0.2	1.0	2.0	7.0	15.0	262.5	–
	ethoxyethoxyethanol	370.0	1.6	3.6	0.1	0.5	1.0	1.5	3.5	7.0	358.5	–
Other aldehydes	tolualdehyde	282.0	0.5	1.4	0.5	0.5	0.5	0.5	0.5	0.5	20.7	–

VOCs: volatile organic compounds; n: number of samples; GM: geometric mean; GSD: geometric standard deviatoin; p10-p90: percentiles.
Ref. value: reference value according to the German Association of Environmental Institutes (AGÖF).
